# First database of invasive crayfish in north-western Italian lakes

**DOI:** 10.3897/BDJ.14.e189983

**Published:** 2026-06-26

**Authors:** Lyudmila Kamburska, Silvia Zaupa, Marco Orlandi, Laura Garzoli, Angela Boggero

**Affiliations:** 1 National Research Council (CNR) - Water Research Institute (IRSA), Verbania, Italy National Research Council (CNR) - Water Research Institute (IRSA) Verbania Italy; 2 National Biodiversity Future Center (NBFC), Palermo, Italy National Biodiversity Future Center (NBFC) Palermo Italy; 3 National Research Council (CNR) - Water Research Institute (IRSA), Taranto, Italy National Research Council (CNR) - Water Research Institute (IRSA) Taranto Italy; 4 National Research Council (CNR) - Water Research Institute (IRSA), Benthic Invertebrates Group (BIG), Verbania, Italy National Research Council (CNR) - Water Research Institute (IRSA), Benthic Invertebrates Group (BIG) Verbania Italy

**Keywords:** GBIF, DwC-A, occurrence, distribution, invasive crayfish, plain lakes, Italy

## Abstract

**Background:**

This data paper presents 367 georeferenced occurrence records of invasive crayfish collected from 17 lakes in north-western Italy between 2017 and 2025. The database comprises four datasets in the standardised Darwin Core Archive format (DwC-A) and is openly accessible through the Global Biodiversity Information Facility (GBIF). We document the datasets, associated metadata, and the projects supporting data acquisition, rather than presenting research findings. All records derive from sampling conducted in submontane lakes in two Italian regions (Piedmont and Lombardy). The lakes represent different origins (fluvial, glacial and tectonic) and typologies, ranging from large, deep systems to small, shallow waterbodies. Two study lakes (Maggiore and Orta) are part of the national Long-Term Ecological Research network (LTER Italy), while the remaining lakes are designated as Special Areas of Conservation, Special Protection Areas or belong to the European Union’s Natura 2000 network under the Birds Directive. By making these data publicly available, this work supports global efforts to mobilise FAIR (Findable, Accessible, Interoperable and Reusable) biodiversity data on invasive species, facilitating regional-scale monitoring and management.

**New information:**

Newly georeferenced records of three invasive crayfish species — *Faxonius limosus*, *Pacifastacus leniusculus* and *Procambarus clarkii* — from 17 lakes in Piedmont and Lombardy (north-western Italy) have been published in GBIF. The data reveal the spatial distribution and relative abundance of these species, supporting ecological research, regional monitoring and the management of freshwater invasions.

## Introduction

Freshwater ecosystems are highly vulnerable to human-mediated species introductions and their associated impacts (Moorhouse and Macdonald 2014, Cuthbert et al. 2021). Invasive crayfish are amongst the most damaging species in Europe, driving biodiversity loss, altering food webs and compromising ecosystem services (O’Hea Miller et al. 2024). Indeed, several introduced non-native crayfish species are listed as species of Union Concern under EU Regulation 1143/2014, with high ecological and economic impacts (Haubrock et al. 2021, Roy et al. 2022, Soto et al. 2023). Significant gaps in the reporting the cost of invasive crayfish, combined with fragmented spatial data, highlight the urgent need for more comprehensive assessments of their true impacts (Diagne et al. 2020, Kouba et al. 2022). Publishing FAIR (Findable, Accessible, Interoperable and Reusable) data facilitates long-term analyses of these impacts (Wilkinson et al. 2016).

We present georeferenced occurrence records for three invasive crayfish species of North American origin from 17 lakes in north-western Italy, published via the Global Biodiversity Information Facility (GBIF). Our data represent the first and, in some cases, the only records of invasive crayfish in these lakes (Boggero et al. 2025a), thereby supporting regional management strategies (Regione Piemonte 2024) and contributing to a broader understanding of crayfish distribution across newly invaded habitats worldwide.

## General description

### Purpose

**Figure 1.**
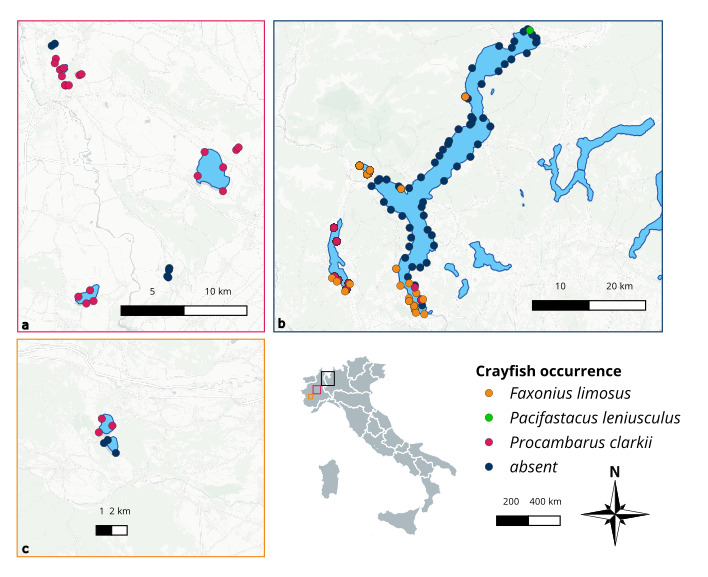
Map of sampled lakes in north-western Italy showing occurrence records of three invasive crayfish species; panel a) 12 small lakes (see Table 1); panel b) Maggiore, Mergozzo, Orta; panel c) Avigliana Grande and Avigliana Piccolo.

Georeferenced occurrence records of invasive crayfish in north-western Italian lakes (Table 1, Fig. 1) were standardised in Darwin Core Archive (DwC-A) format and made available through GBIF. This database is increasing the visibility of our data and it could be linked to the Invasive Species Specialist Group (2026) and to the Global Register of Introduced and Invasive Species (Carnevali et al. 2020). Updated data on crayfish distribution in the Piedmont and Lombardy provide a basis for regional management strategies.

## Project description

### Title

Occurrence and distribution of invasive crayfish species in north-western Italian lakes

### Personnel

Angela Boggero, Carlo Croci, Laura Garzoli, Lyudmila Kamburska, Marco Orlandi, Daniele Paganelli, Denise Schiavetta, Silvia Zaupa

### Study area description

The geographic coverage of the four datasets includes 17 lakes in north-western Italy (Fig. 1). The main characteristics of these lakes are reported in Table 1.

### Design description

We investigated the occurrence of introduced crayfish in several north-western Italian lakes during the period 2017 – 2025. Three invasive crayfish species were recorded during these investigations: *Faxonius limosus* (Rafinesque, 1817), *Pacifastacus leniusculus* (Dana, 1852) and *Procambarus clarkii* (Girard, 1852) (Fig. 2). Prior to 2017, *F. limosus* was formerly classified as *Orconectes limosus* (*Crandall and De Grave 2017*).

**Figure 3.**
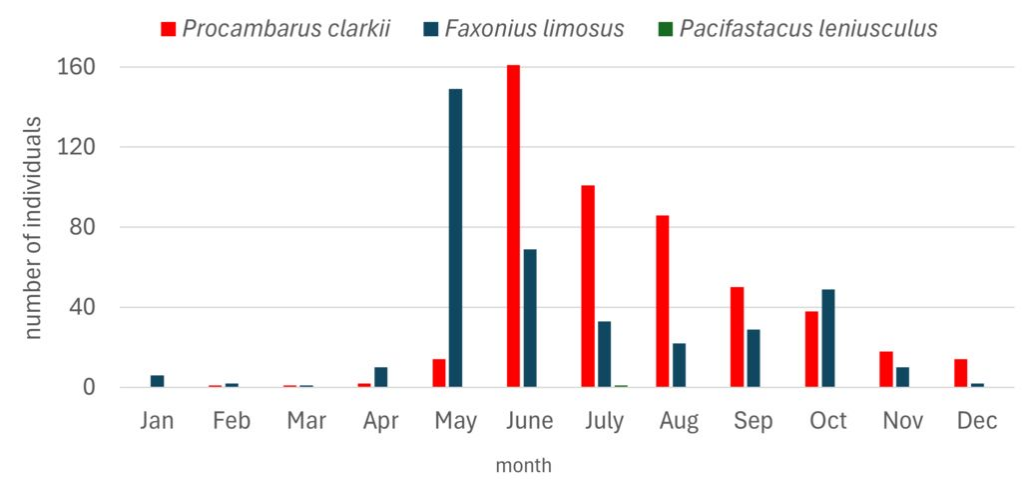
Monthly total number of individuals of three invasive crayfish species across Lakes Maggiore, Mergozzo and Orta.

All data were digitised and organised into four datasets, corresponding to different lakes, sampling periods, personnel involved and funding. Finally, 367 georeferenced records were published through GBIF. The most recent dataset reports occurrence records from 17 lakes sampled between June and October 2025. The other three datasets cover single lakes (Maggiore, Mergozzo, Orta), sampled between 2017 and 2022, and report the number of organisms observed. Total abundance exhibited a clear seasonal pattern, with *F. limosus* and *P. clarkii* reaching peak densities in the summer–autumn period (Fig. 3). The predominance of *P. clarkii* in summer further supports its classification as a warm-water invader (Haubrock et al. 2019). *Pacifastacus leniusculus* was recorded only in July 2018, in the northernmost part of Lake Maggiore (Swiss territory).

### Funding

Funding sources for the sampling campaigns and personnel, across different time periods: (1) National Biodiversity Future Center funded under the National Recovery and Resilience Plan (NRRP), Mission 4 Component 2 Investment 1.4- Call for tender No. 3138, 16/12/2021, rectified by Decree No. 3175, 18/12/2021 of the Italian Ministry of University and Research funded by the European Union – NextGenerationEU (Project code CN_00000033, Concession Decree No. 1034, 17/06/2022); (2) CUSIO 2030, Conservation and requalification measures of common reed *Ph. australis*’s habitats of Lake Orta (CUP B53C23000930007); (3) International Commission for the Protection of Italian-Swiss Waters (CIPAIS – Cooperation agreement 769/2019).

## Sampling methods

### Sampling description

Sampling sites were selected with respect to sediment type and vegetation cover considered suitable habitat for crayfish, where the occurrence of crayfish was previously confirmed or suspected. The number of sampling sites per lake was proportional to surface area (e.g. 4 in Lake Mergozzo; 6 in Lake Orta). At each site, 13 interconnected traps (2 – 3 m apart) were deployed on the shallow lake bottom overnight for approximately 12 hours, coinciding with peak crayfish activity. Each cylindrical trap (30 cm × 60 cm, mesh size 1 cm²) was baited with 20 g of cat food to attract crayfish. The number of crayfish captured per trap was recorded to calculate the Catch Per Unit Effort (CPUE) for each sampling site, defined as the total catch divided by the sampling effort (number of traps × night). All native species caught incidentally were immediately released (Boggero et al. 2023).

### Quality control

Geographic coordinates were verified using Google Maps. Specimens were identified to species level by taxonomic experts using identification keys (Souty-Grosset et al. 2006); molecular analyses were performed when necessary to confirm doubtful cases.

## Geographic coverage

### Description

Europe, Italy, Piedmont, Lombardy (Fig. 1). Dataset for Lake Maggiore includes data also from sampling sites in Switzerland.

## Taxonomic coverage

### Description

Three species, native to North America: *Faxonius limosus* (Rafinesque, 1817) from the Atlantic watershed of North America; *Pacifastacus leniusculus* (Dana, 1852) from the north-western USA and *Procambarus clarkii* (Girard, 1852) from southern USA and north-eastern Mexico (Ion 2024).

### Taxa included

**Table:** 

Rank	Scientific Name	
kingdom	Animalia	
phylum	Arthropoda	
class	Malocostraca	
order	Decapoda	
family	Cambaridae	
genus	Procambarus	
species	*Procambarus clarkii* (Girard, 1852)	red swamp crayfish
genus	Faxonius	
species	*Faxonius limosus* (Rafinesque, 1817)	spiny-cheek crayfish
family	Astacidae	
genus	Pacifastacus	
species	*Pacifastacus leniusculus* (Dana, 1852)	signal crayfish

## Traits coverage

This database has contributed to several peer-reviewed publications. Garzoli et al. (2020) analysed the biometry and habitat preferences of the spiny-cheek crayfish *F. limosus* in Lake Maggiore, highlighting also the occurrence of *P. leniusculus*, limited to the northern part of the Lake (Swiss territory). Boggero et al. (2023) documented *F. limosus* (Rafinesque, 1817) in Lakes Orta and Mergozzo, calculating also the CPUE. The study analysed sex ratio and morphological traits across subalpine Lakes Maggiore, Orta and Mergozzo. Kamburska et al. (2024) reported a new morphotype of *P. clarkii* with atypical marbled colouration, morphologically similar to *P. virginalis*, in Lake Orta. Boggero et al. (2025a) documented the expansion of invasive crayfish in several Piedmont lakes, their habitat preferences, discussing CPUE and different hypotheses for "invasion history" of the species.

## Temporal coverage

**Formation period:** August 7, 2017 - July 12, 2018; June 15, 2021 - May 18, 2022; June 8, 2021 - May 17, 2022; June 12, 2025 - October 1, 2025.

### Notes

Formation period of each dataset is provided. A unique Digital Object Identifier (DOI) is assigned to each dataset within GBIF.

## Usage licence

### Usage licence

Creative Commons Public Domain Waiver (CC-Zero)

### IP rights notes

data records in GBIF under CC BY-NC 4.0

## Data resources

### Data package title

Invasive crayfish occurrence database in north-western Italian lakes

### Number of data sets

4

### Data set 1.

#### Data set name

Invasive crayfish occurrence in the subalpine Lake Maggiore (NW Italy) in 2017- 2018

#### Data format

csv

#### Download URL

https://doi.org/10.15468/a99fkj

#### Data format version

1.12

#### Description

In total, 80 occurrence records of *P. clarkii*, *F. limosus* and *P. leniusculus* in the subalpine Lake Maggiore from April 2017 to July 2018 from 75 sampling points (Fig. 1b) are included in this dataset (Boggero et al. 2026a). *Pacifastacus leniusculus* was reported for the first time (Boggero et al. 2018, Garzoli et al. 2020). The Lake is systematically monitored by the International Commission for the Protection of the Italian-Swiss Waters (CIPAIS). However, *P. clarkii* and *P. leniusculus* were recorded rarely, while *F. limosus* dominated especially in May (Table 2). The mean CPUE of *F. limosus* was 1.14 (Boggero et al. 2023).

**Data set 1.** 

Column label	Column description
occurrenceID	Unique number corresponding to specific occurrence.
basisOfRecord	The specific nature of the data record.
eventDate	The date-time or interval during which an Event occurred.
year	The four-digit year in which the Event occurred.
month	The integer month in which the Event occurred.
day	The integer day of the month on which the Event occurred.
scientificName	The full scientific name, with authorship and date information if known.
higherClassification	A list of taxa names terminating at the rank immediately superior to the referenced Taxon.
kingdom	The full scientific name of the kingdom in which the Taxon is classified.
phylum	The full scientific name of the phylum or division in which the scientific name is classified.
class	The full scientific name of the class in which the Taxon is classified.
order	The full scientific name of the order in which the scientificName is classified.
family	The full scientific name of the family in which the scientificName is classified.
genus	The full scientific name of the genus in which the Taxon is classified.
specificEpithet	The name of the first or species epithet of the scientificName.
taxonRank	The taxonomic rank of the most specific name in the scientificName.
degreeOfEstablishment	The degree to which an Organism survives, reproduces and expands its range at the given place and time.
identifiedBy	A list (concatenated and separated) of names of people, groups or organisations who assigned the Taxon to the subject.
decimalLatitude	The geographic latitude (in decimal degrees, using the spatial reference system given in geodeticDatum) of the geographic centre of a terms Location.
decimalLongitude	The geographic longitude (in decimal degrees, using the spatial reference system given in geodetic Datum) of the geographic centre of a terms Location.
geodeticDatum	The ellipsoid, geodetic datum or spatial reference system (SRS) upon which the geographic coordinates given in decimalLatitude and decimalLongitude are based.
higherGeography	A list (concatenated and separated) of geographic names less specific than the information captured in the locality term.
continent	The name of the continent in which the terms Location occurs.
country	The name of the country or major administrative unit in which the Location occurs.
countryCode	The standard code for the country in which the terms Location occurs.
stateProvince	The specific description of the place.
county	The name of the country or major administrative unit in which the terms location occurs.
locality	The specific description of the place.
language	A language of the resource.
licence	A legal document giving official permission to do something with the resource.
associatedReferences	A list of identifiers (publication, bibliographic reference, global unique identifier, URI) of literature associated with the Occurrence.
institutionCode	The name in use by the institution having custody of the object(s) or information referred to in the record.
institutionID	An identifier for the institution having custody of the object(s) or information referred to in the record.
recordedBy	A person, group or organisation responsible for recording the original Occurrence.
organismQuantity	A number or enumeration value for the quantity of Organisms.
organismQuantityType	The type of quantification system used for the quantity of Organisms.
occurrenceStatus	A statement about the presence or absence of a taxon at a Location.

### Data set 2.

#### Data set name

Invasive crayfish occurrence in the subalpine Lake Orta (NW Italy) in 2021- 2022

#### Data format

csv

#### Download URL

https://doi.org/10.15468/tv6dcr

#### Data format version

1.16

#### Description

Dataset includes 186 occurrence records of *F. limosus* and *P. clarkii* at six sampling sites in Lake Orta collected monthly from June 2021 to May 2022 (Boggero et al. 2026b). The co-existence of these two invasive species reveals that *P. clarkii* accounts for over 80% of the total abundance from May to December, dominating *F. limosus* (Table 2). *Procambarus clarkii* was reported for the first time in the Lake in 2010 (Piscia et al. 2011a, Piscia et al. 2011b), while *F. limosus* only during this survey (Boggero et al. 2023). The mean CPUE of *F. limosus* was 1.03, while CPUE of *P. clarkii* was 0.78. However, mean CPUE of both species was significantly altered in 2025 (Boggero et al. 2025).

**Data set 2.** 

Column label	Column description
occurrenceID	Unique number corresponding to specific occurrence.
basisOfRecord	The specific nature of the data record.
eventDate	The date-time or interval during which an Event occurred.
year	The four-digit year in which the Event occurred.
month	The integer month in which the Event occurred.
day	The integer day of the month on which the Event occurred.
scientificName	The full scientific name, with authorship and date information if known.
higherClassification	A list of taxa names terminating at the rank immediately superior to the referenced Taxon.
kingdom	The full scientific name of the kingdom in which the Taxon is classified.
phylum	The full scientific name of the phylum or division in which the scientific name is classified.
class	The full scientific name of the class in which the Taxon is classified.
order	The full scientific name of the order in which the scientificName is classified.
family	The full scientific name of the family in which the scientificName is classified.
genus	The full scientific name of the genus in which the Taxon is classified.
specificEpithet	The name of the first or species epithet of the scientificName.
degreeOfEstablishment	The degree to which an Organism survives, reproduces and expands its range at the given place and time.
taxonRank	The taxonomic rank of the most specific name in the scientificName.
decimalLatitude	The geographic latitude (in decimal degrees, using the spatial reference system given in geodeticDatum) of the geographic centre of a terms Location.
decimalLongitude	The geographic longitude (in decimal degrees, using the spatial reference system given in geodetic Datum) of the geographic centre of a terms Location.
geodeticDatum	The ellipsoid, geodetic datum or spatial reference system (SRS) upon which the geographic coordinates given in decimalLatitude and decimalLongitude are based.
higherGeography	A list (concatenated and separated) of geographic names less specific than the information captured in the locality term.
continent	The name of the continent in which the terms Location occurs.
country	The name of the country or major administrative unit in which the Location occurs.
countryCode	The standard code for the country in which the terms Location occurs.
stateProvince	The specific description of the place.
county	The name of the country or major administrative unit in which the terms location occurs.
locality	The specific description of the place.
language	A language of the resource.
licence	A legal document giving official permission to do something with the resource.
associatedReferences	A list of identifiers (publication, bibliographic reference, global unique identifier, URI) of literature associated with the Occurrence.
institutionID	An identifier for the institution having custody of the object(s) or information referred to in the record.
institutionCode	The name in use by the institution having custody of the object(s) or information referred to in the record.
organismQuantity	A number or enumeration value for the quantity of Organisms.
organismQuantityType	The type of quantification system used for the quantity of Organisms.
occurrenceStatus	A statement about the presence or absence of a Taxon at a Location.

### Data set 3.

#### Data set name

Occurrence of invasive *Faxonius limosus* in the subalpine Lake Mergozzo (NW-Italy) in 2021-2022

#### Data format

csv

#### Download URL

https://doi.org/10.15468/f4jv5n

#### Data format version

1.10

#### Description

This dataset includes 54 occurrence records of *F. limosus* collected monthly from June 2021 to May 2022 at four sampling sites in Lake Mergozzo (Kamburska et al. 2026b). These activities led to the first record of the species in the Lake (Boggero et al. 2023). Its abundance peaked between June and October 2021, followed by occasional records from March to May 2022 (Table 2). The mean CPUE of *F. limosus* was 1.08 (Boggero et al. 2023). Only *F. limosus* has been recorded and no other crayfish species detected.

**Data set 3.** 

Column label	Column description
occurrenceID	Unique number corresponding to specific occurrence.
basisOfRecord	The specific nature of the data record.
eventDate	The date-time or interval during which an Event occurred.
year	The four-digit year in which the Event occurred.
month	The integer month in which the Event occurred.
day	The integer day of the month on which the Event occurred.
scientificName	The full scientific name, with authorship and date information if known.
higherClassification	A list of taxa names terminating at the rank immediately superior to the referenced Taxon.
kingdom	The full scientific name of the kingdom in which the Taxon is classified.
phylum	The full scientific name of the phylum or division in which the scientific name is classified.
class	The full scientific name of the class in which the Taxon is classified.
order	The full scientific name of the order in which the scientificName is classified.
family	The full scientific name of the family in which the scientificName is classified.
genus	The full scientific name of the genus in which the Taxon is classified.
specificEpithet	The name of the first or species epithet of the scientificName.
taxonRank	The taxonomic rank of the most specific name in the scientificName
decimalLatitude	The geographic latitude (in decimal degrees, using the spatial reference system given in geodeticDatum) of the geographic centre of a terms Location.
decimalLongitude	The geographic latitude (in decimal degrees, using the spatial reference system given in geodeticDatum) of the geographic centre of a terms Location.
geodeticDatum	The ellipsoid, geodetic datum or spatial reference system (SRS) upon which the geographic coordinates given in decimalLatitude and decimalLongitude are based.
higherGeography	A list (concatenated and separated) of geographic names less specific than the information captured in the locality term.
continent	The name of the continent in which the terms Location occurs.
country	The name of the country or major administrative unit in which the Location occurs.
countryCode	The standard code for the country in which the terms Location occurs.
stateProvince	The name of the next smaller administrative region than country (state, province, canton, department, region etc.) in which the term Location occurs.
county	The name of the country or major administrative unit in which the terms Location occurs.
locality	The specific description of the place.
language	A language of the resource.
licence	A legal document giving official permission to do something with the resource.
associatedReferences	A list of identifiers (publication, bibliographic reference, global unique identifier, URI) of literature associated with the Occurrence.
institutionID	An identifier for the institution having custody of the object(s) or information referred to in the record.
institutionCode	The name in use by the institution having custody of the object(s) or information referred to in the record.
organismQuantity	A number or enumeration value for the quantity of Organisms.
organismQuantityType	The type of quantification system used for the quantity of Organisms.
occurrenceStatus	A statement about the presence or absence of a Taxon at a Location.
degreeOfEstablishment	The degree to which an Organism survives, reproduces and expands its range at the given place and time.

### Data set 4.

#### Data set name

Invasive crayfish occurrence in Piedmont plain lakes (NW Italy)

#### Data format

csv

#### Download URL

https://doi.org/10.15468/knwyc9

#### Data format version

1.8

#### Description

This dataset reports 47 occurrenceStatus (present/absent) records of *F. limosus* and *P. clarkii* from 17 lakes sampled once between late June and early October 2025 (Kamburska et al. 2026a). In seven of the 17 lakes, crayfish species were absent (Table 3), while *P. clarkii* was reported for the first time in Pistono, San Michele and Sirio, expanding our knowledge of crayfish distribution in Piedmont freshwaters (Boggero et al. 2025). Thus, mean CPUE of *P. clarkii* varied across the lakes: ≥1.5 individuals in Candia and Pistono, 0.5–1.5 in Bertignano, Campagna, and San Michele, and did not exceed 0.5 in Avigliana Grande, Maggiore, Orta, Sirio, and Viverone.

*Faxonius limosus* was recorded only in Lakes Orta and Maggiore, with mean CPUE of 1.27 in Lake Maggiore and 0.05 in Lake Orta (Boggero et al. 2025), both much lower than those recorded during 2021–2022 (Boggero et al. 2023). It was hypothesised the occurrence of non-native crayfish is influenced by hydrological connectivity, predator–prey interactions and illegal activities (Boggero et al. 2025).

**Data set 4.** 

Column label	Column description
occurrenceID	Unique number corresponding to specific occurrence.
basisOfRecord	The specific nature of the data record.
eventDate	The date-time or interval during which an Event occurred.
year	The four-digit year in which the Event occurred.
month	The integer month in which the Event occurred.
day	The integer day of the month on which the Event occurred.
scientificName	The full scientific name, with authorship and date information if known.
higherClassification	A list of taxa names terminating at the rank immediately superior to the referenced Taxon.
kingdom	The full scientific name of the kingdom in which the Taxon is classified.
phylum	The full scientific name of the phylum or division in which the scientific name is classified.
class	The full scientific name of the class in which the Taxon is classified.
order	The full scientific name of the order in which the scientificName is classified.
family	The full scientific name of the family in which the scientificName is classified.
genus	The full scientific name of the genus in which the Taxon is classified.
specificEpithet	The name of the first or species epithet of the scientificName.
taxonRank	The taxonomic rank of the most specific name in the scientificName.
occurrenceStatus	A statement about the presence or absence of a taxon at a Location.
decimalLatitude	The geographic latitude (in decimal degrees, using the spatial reference system given in geodeticDatum) of the geographic centre of a terms Location.
decimalLongitude	The geographic longitude (in decimal degrees, using the spatial reference system given in geodetic Datum) of the geographic centre of a terms Location.
geodeticDatum	The ellipsoid, geodetic datum or spatial reference system (SRS) upon which the geographic coordinates given in decimalLatitude and decimalLongitude are based.
higherGeography	A list (concatenated and separated) of geographic names less specific than the information captured in the locality term.
continent	The name of the continent in which the terms Location occurs.
country	The name of the country or major administrative unit in which the terms Location occurs.
countryCode	The standard code for the country in which the terms Location occurs.
stateProvince	The name of the next smaller administrative region than country (state, province, canton, department, region etc.) in which the terms Location occurs.
county	The name of the country or major administrative unit in which the terms location occurs.
waterBody	The name of the waterBody in which the terms Location occurs.
eventRemarks	Comments or notes about the Event.
maximumElevationInMetres	The upper limit of the range of elevation (altitude, above sea level) in metres.
maximumDepthInMetres	The greater depth of a range of depth below the local surface, in metres.
habitat	A category or description of the habitat in which the Event occurred.
language	A language of the resource.
eventType	The nature of the Event.
licence	A legal document giving official permission to do something with the resource.
institutionID	An identifier for the institution having custody of the object(s) or information referred to in the record.
ownerInstitutionCode	The name (or acronym) in use by the institution having ownership of the object(s) or information referred to in the record.
recordedBy	A list (concatenated and separated) of names of people responsible for recording the original Occurrence.
recordedByID	A list (concatenated and separated) of the globally unique identifier for the person, people, groups or organisations responsible for recording the original Occurrence.
references	A related resource that is referenced, cited or otherwise pointed to by the described resource.

## Figures and Tables

**Figure 2a.**
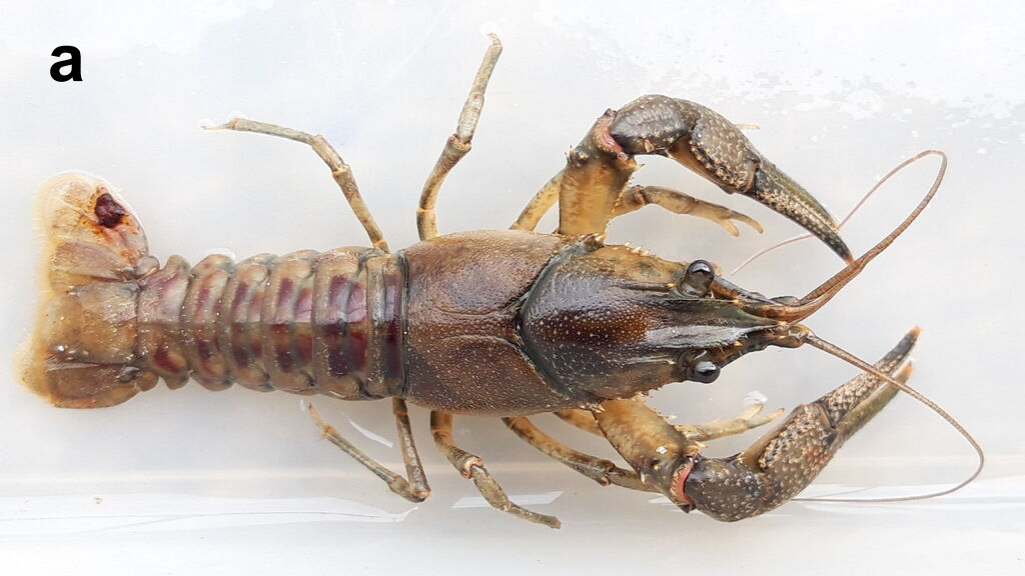
Faxonius limosus

**Figure 2b.**
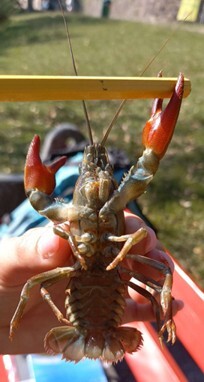
Pacifastacus leniusculus

**Figure 2c.**
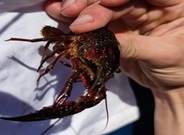
Procambarus clarkii

**Table 1.** Sampled lakes: altitude (m a.s.l.), WGS84 coordinates (decimal degrees, DD), surface area (km²), maximum depth (m); Panels a, b and c refer to lake location in Fig. 1.

**Panel**	**Lake**	**Altitude** **m a.s.l**.	**WGS84 (DD)**		**Surface** **area** **km²**	**Max depth** **m**
			**Latitude N**	**Longitude E**		
a	Alice Superiore	575	45.46222	7.79528	0.1	11
a	Bertignano	377	45.43222	8.06222	0.1	11
a	Campagna	238	45.48389	7.89500	0.1	5
a	Candia	226	45.32361	7.91194	1.5	8
a	Maglione	251	45.34528	7.99556	0.1	2
a	Meugliano	715	45.47667	7.78972	0.03	11
a	Moncrivello	263	45.34000	7.99222	0.03	2
a	Nero	342	45.50472	7.87333	0.1	27
a	Pistono	280	45.49278	7.87444	0.1	16
a	San Michele	238	45.14361	7.88778	0.1	19
a	Sirio	266	45.48694	7.88389	0.3	44
a	Viverone	230	45.41639	8.03556	5.7	50
b	Maggiore	193	46.09806	8.71472	212.5	372
b	Mergozzo	204	45.95556	8.46667	1.8	73
b	Orta	290	45.81722	8.40667	18.2	143
c	Avigliana Grande	352	45.06583	7.38694	0.9	28
c	Avigliana Piccolo	356	45.05361	7.39167	0.6	12

**Table 2.** Monthly cumulative and total annual numbers (n) of captured crayfish by species and lake (dataset).

**Month Lake (dataset)**	**Jan**	**Feb**	**Mar**	**Apr**	**May**	**Jun**	**Jul**	**Aug**	**Sep**	**Oct**	**Nov**	**Dec**	**n**
**Lake Maggiore (2017-2018)**														
*F. limosus*				9	146	42	6	0		37	0		240
*P. leniusculus*				0	0	0	1	0		0	0		1
*P. clarkii*				0	0	0	1	0		0	0		1
**Lake Orta (2021-2022)**														
*F. limosus*	2	1	1	1	3	26	21	13	13	3	2	0	86
*P. clarkii*	0	1	1	2	14	161	100	86	50	38	18	14	485
**Lake Mergozzo (2021-2022)**														
*F. limosus*	4	1	0	0	0	1	6	9	16	9	8	2	56

**Table 3.** Occurrence status of invasive crayfish in lakes sampled between June and October 2025; *FL (Faxonius limosus)*, *PC (Procambarus clarkii) -* present, x - absent.

**n**.	**Lake**	**Crayfish occurrence**
1	Alice Superiore	x
2	Bertignano	*PC*
3	Campagna	*PC>*
4	Candia	*PC>*
5	Maglione	x
6	Meugliano	x
7	Moncrivello	x
8	Nero	x
9	Pistono	*PC>*
10	San Michele	*PC>*
11	Sirio	*PC>*
12	Viverone	*PC>*
13	Maggiore	*FL, PC*
14	Mergozzo	x
15	Orta	*FL, PC*
16	Avigliana Grande	*PC>*
17	Avigliana Piccolo	x
